# CPEO and Mitochondrial Myopathy in a Patient with* DGUOK* Compound Heterozygous Pathogenetic Variant and mtDNA Multiple Deletions

**DOI:** 10.1155/2019/5918632

**Published:** 2019-03-06

**Authors:** V. Montano, C. Simoncini, Cassi L. Calì, A. Legati, G. Siciliano, M. Mancuso

**Affiliations:** ^1^Department of Clinical and Experimental Medicine, Neurological Clinic, University of Pisa, Pisa, Italy; ^2^Department of Hand Surgery and Reconstructive Microsurgery, University of Pisa, Pisa, Italy; ^3^Unit of Medical Genetics and Neurogenetics, Fondazione IRCCS Istituto Neurologico “C. Besta”, Milan, Italy

## Abstract

The classic features of deoxyguanosine kinase (*DGUOK*) deficiency are infantile onset hepatic failure with nystagmus and hypotonia; mitochondrial DNA studies on affected tissue reveal mitochondrial DNA depletion. Later, it has been shown that the mutations in the same gene may present with adult-onset mitochondrial myopathy and mitochondrial DNA multiple deletions in skeletal muscle. Here we report the case of a 42-year-old Italian woman presenting with a chronic progressive external ophthalmoplegia and myopathy with mtDNA multiple deletions and the compound heterozygous c.462T>A (p.Asn154Lys) and c.707+2T>G pathogenic variants in* DGUOK*.

## 1. Introduction

Mitochondrial diseases are multisystem disorders caused by mutation in mitochondrial DNA (mtDNA) or in nuclear DNA (nDNA). Many proteins encoded by nDNA control the replication of mtDNA and the pull of the mtDNA nucleotides necessary for replication; the deficit of these proteins causes mtDNA depletion or multiple deletions or both [1]. The deoxyguanosine kinase (dGK), encoded by* DGUOK *gene (chromosome 2p13, MIM 601465), is an enzyme that catalyzes the phosphorylation of purine deoxyribonucleosides into the corresponding nucleotides; it is necessary for the maintenance of mitochondrial deoxyribonucleoside triphosphate (dNTP) pools and its deficiency results in impaired synthesis of mitochondrial dNTPs, the building blocks for mtDNA, leading to decreased mtDNA amount and mtDNA depletion. The phenotypes associated with the mutation of those gene are most frequently mtDNA depletion syndrome 3 (hepatocerebral type, phenotype MIM number 251880), noncirrhotic portal hypertension (MIM number 617068), and, less frequently, chronic progressive external ophthalmoplegia (cPEO) with mtDNA deletions (MIM number 617070) [2–4].

Here we describe a case of cPEO and myopathy with mtDNA multiple deletions and the compound heterozygous c.462T>A (p.Asn154Lys) and c.707+2T>G pathogenic variants in* DGUOK* gene.

## 2. Case Report

**Figure 1:**
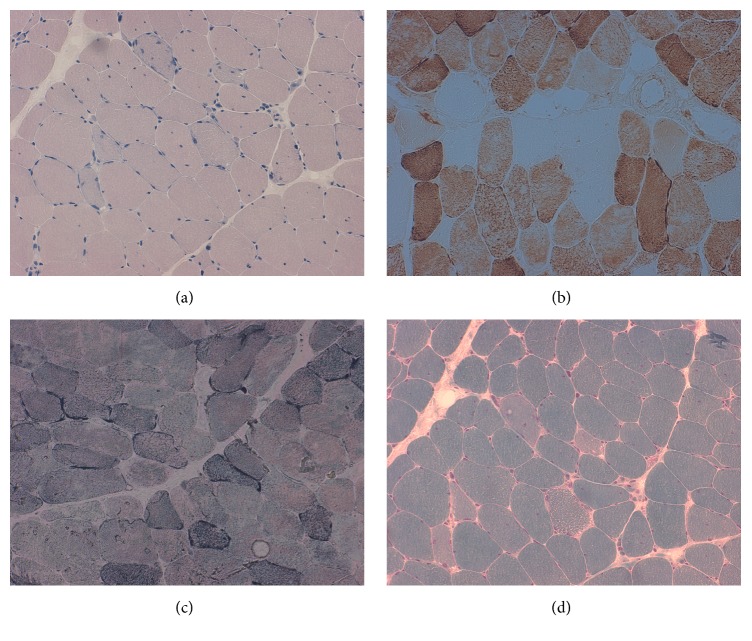
Muscle biopsy: (a) hematoxylin eosin; (b) staining for cytochrome oxidase; (c) succinate dehydrogenase (SDH) staining; (d) Gömöri trichrome stain.

**Figure 2:**
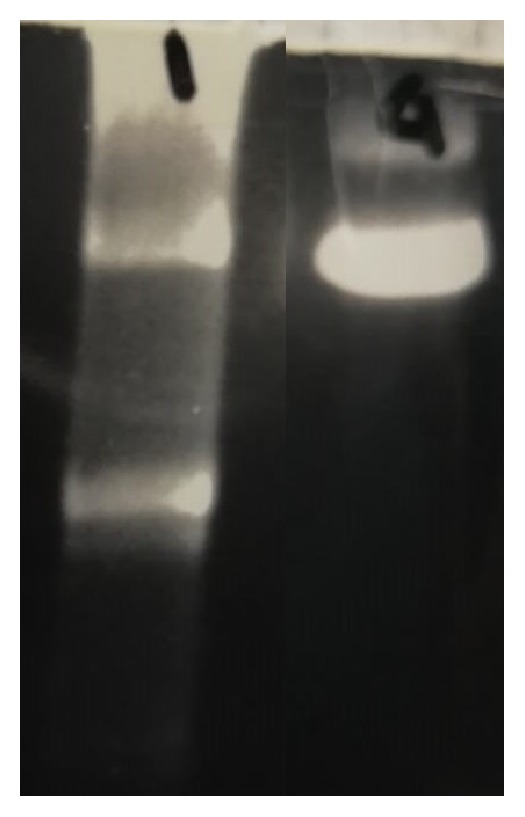
Long-PCR analysis of mitochondrial DNA in muscle tissue: on the left, the test performed on our patient, which shows multiple deletions of mtDNA, and, on the right, a normal control.

A 42-year-old Italian woman arrived to our attention for a two-year history of eyelid ptosis, ophthalmoparesis, dysphagia, exercise intolerance, and myalgia. She presented mild hyperCKemia (243U/L). Patient's parents were not consanguineous. She had no family history of neurological disorder. In the past, she has suffered of anxiety disorder. Neurological examination showed mild proximal weakness of lower and upper limbs, weakness of facial muscles, bilateral eyelid ptosis, and ophthalmoparesis. Forearm ischaemic test revealed basal hyperlactacidemia (25 mg/dL, reference value: 4,5-19,8 mg/dl), increased production, and delay in the recovery of lactate. Electromyography showed a myopathic pattern. Echocardiogram was normal but the ergospirometric test showed a functional limitation; spirometry was normal but maximal inspiratory pressure (MIP) and maximal expiratory pressure (MEP) were reduced (MIP=4,1 kPA, n.v. >7,61, MEP 5,17 kPA, n.v. >10,2). Muscle biopsy revealed ragged red and ragged blue fibers and COX negative fibers (Figure 1). Sequencing of the entire mtDNA from muscle was normal. Long-PCR analysis, in muscle tissue, showed mtDNA multiple deletions (Figure 2), and next-generation sequencing (NGS) analysis detected two* DGOUK* compound heterozygous mutations: the known pathogenetic variant c.462T>A (p.Asn154Lys) and a new variant of the donor splice site of intron 5 c.707+2T>G, predicted to alter the splicing on Human Splicing Finder (http://www.umd.be/HSF3/HSF.shtml) and thus considered pathogenetic. While we could not test their parents, two asymptomatic siblings harbored the c.707+2T>G pathogenetic variant. Since we could not test the parents, it is possible to assume that the two variants were inherited in an autosomal recessive manner in the patient; however, it is equally possible one arose de novo.

## 3. Discussion

Mitochondrial disorders are the largest group of inherited metabolic disorders and the commonest adult forms of inherited neurological disorders that arise as a result of dysfunction of the mitochondrial respiratory chain and, consequently, deficient energy production [5]. mtDNA encodes for part of its machinery, while most of the mitochondrial proteins are encoded by the nDNA. Accordingly, mitochondrial disorders can be due to mutation of genes encoded by either nuclear DNA or mtDNA. A number of nuclear-encoded factors control mtDNA replication and the maintenance of qualitative and quantitative mtDNA integrity. The impaired cross-talk between the two genomes gives rise to so-called nuclear-mitochondrial intergenomic communication disorders, which result in loss or instability of the mitochondrial genome and impaired maintenance of qualitative and quantitative mtDNA integrity: multiple deletions, depletion, or combinations of the two in critical tissues are “hallmarks” of disease conditions arising from altered communication between these two genomes [6]. The mtDNA depletion syndromes are usually clinically classified as myopathic (*TK2* [thymidine kinase 2] mutation), encephalomyopathic (mutation in* SUCLA2* [Succinate-Coa Ligase, Adp-Forming, Beta Subunit],* SUCLG1* [Succinate-Coa Ligase, Alpha Subunit] and* RRM2B* [Ribonucleotide Reductase M2 B Subunit]), hepatocerebral (due to mutation in DGUOK,* MPV17* [Mitochondrial Inner Membrane Protein],* POLG *[DNA Polymerase Subunit Gamma] and* TWNK* [Twinkle mtDNA Helicase]), or neurogastrointestinal (mutations in TYMP [Thymidine Phosphorylase]) [7, 8].

The other hallmarks of mtDNA instability are mtDNA multiple deletions, which have been shown to accumulate even in the aging of postmitotic tissue [9, 10]. Multiple deletions due to nuclear gene mutation in* POLG1*,* POLG2*,* C10Orf2*,* SLC25A4*,* RRM2B*,* OPA1*, and many others may present with clinical phenotypes ranging from chronic external ophthalmoplegia to multisystem involvement including ataxia, parkinsonism, sensory axonal neuropathy, and optic atrophy [11–13].

The deoxyguanosine kinase (dGK), encoded by* DGUOK* gene, plays an important role in maintaining of mitochondrial deoxyribonucleoside triphosphate (dNTP) pools; its deficiency results in impaired synthesis of mitochondrial dNTPs.

*DGUOK* mutations were firstly associated with mtDNA depletion syndrome by Mandel et al. in 2001 [2]. Classical features associated with* DGUOK* deficiency are lactic acidosis, hypoglycemia, hepatic disease leading to liver failure and neurologic dysfunction with developmental regression, and typical rotary nystagmus or opsoclonus [14]. In 2012, Ronchi et [3] al. identified with NGS sequencing five patients with multiple deletions and autosomal recessive mutations in the* DGUOK* gene, with heterogeneous neuromuscular presentations ranging from PEO with limb girdle weakness to lower motor neuron disease with bulbar symptoms; interestingly, extra muscle involvement was reported (cataract and sensorineural deafness) and only two of the five patients presented hepatic involvement [3]. Buchaklian in the same year reported a patient with mitochondrial myopathy, mtDNA depletion, and a novel* DGUOK* mutation. Vilarinho in 2016 identified with whole exome sequencing the p.Asn46Ser* DGUOK* missense mutation in childhood or infancy onset portal hypertension of indeterminate etiology [15]; multisystem involvement with CPEO, mitochondrial myopathy, parkinsonism, and mtDNA deletions has recently been described by Caporali [16].

Our patient harbored the well-known pathogenic c.462T>A (p.Asn154Lys) pathogenic variant, already described as pathogenic [3, 8, 14], and a new variant not reported in the available databases (HGMD and ClinVar), predicted to be pathogenic altering the wild-type donor site.

We believe that the* DGUOK *mutations are pathogenic for the following reasons: (i) the phenotype of our patient is suggestive of a mitochondrial disease; (ii) all genes causing nuclear-mitochondrial intergenomic communication disorders have been screened in our patient, together with the entire mtDNA, with no mutations detected besides the ones in* DGUOK; *(iii) we have detected mtDNA multiple deletions, which are commonly observed as a consequence of* DGUOK* mutations.

Our report expands the* DGUOK *mutations causing mitochondrial myopathy and reinforces the concept that* DGUOK* mutations should be screened in adulthood patients with mitochondrial myopathy.
